# Increased Prevalence of Antimicrobial Resistance in* Vibrio cholerae* in the Capital and Provincial Areas of Zambia, January 2023–February 2024

**DOI:** 10.4269/ajtmh.24-0558

**Published:** 2025-01-21

**Authors:** Thandiwe Msipu Phiri, Tadatsugu Imamura, Peter Chibale Mwansa, Ilunga Mathews, Frazer Mtine, Jedidiah Chanda, Mulenga Salasini, Takanori Funaki, Kapona Otridah, Kunda Musonda, Roma Chilengi

**Affiliations:** ^1^ Department of National Public Health Laboratory Services, Zambia National Public Health Reference Laboratory, Lusaka, Zambia;; ^2^ Japan International Cooperation Agency, Tokyo, Japan;; ^3^ National Center for Child Health and Development, Tokyo, Japan;; ^4^ Zambia National Public Health Institute, Lusaka, Zambia

## Abstract

Zambia experienced the largest cholera epidemic in the country’s history in 2023–2024; however, the antimicrobial susceptibility profile of *Vibrio cholerae* during the epidemic is unknown. A total of 2,384 stool samples were collected from suspected cholera cases in Eastern, Lusaka, and Luapula provinces in Zambia from January 2023 to March 2024. Among them, 549 (23.5%, *n* = 549 of 2,341) were culture positive for *V. cholerae* O1, and antimicrobial susceptibility results were available for 431 (78.5%, *n* = 431 of 549). Sensitivity for tetracycline was 84.5% (*n* = 316 of 374) in Lusaka, whereas it was 100% in Eastern and Luapula provinces. Isolates resistant to azithromycin were found only in Lusaka (1.6%, *n* = 1 of 61). Sensitivity for ciprofloxacin was 81.8% (*n* = 260 of 318) in Lusaka province, whereas it was 100% in other provinces. Our results suggested an increased prevalence of antimicrobial resistance in* V. cholerae* against the first- and second-line antibiotic treatments, particularly in the capital. Careful monitoring of the regional antibiogram is warranted.

Cholera is an acute diarrheal disease caused by *Vibrio cholerae* infection. Increased prevalence of multidrug-resistant strains has been a public health concern in Africa as antibiotics are recommended treatment options for severely ill patients in addition to rehydration therapy.^1^^,^^2^ Zambia has experienced over 30 cholera outbreaks and over 10,000 cases cumulatively between 1977 and 2019.^3^ The outbreak that occurred in the capital, Lusaka, between October 2017 and May 2018 caused more than 5,000 cases and resulted in 90 fatal cases.^4^ This called for a large-scale public health response, which was coordinated by the Ministry of Health through the Zambia National Public Health Institute (ZNPHI).^4^

In 2023–2024, cholera cases were reported from known cholera hot spot districts (e.g., Nchelenge and Lusaka districts) and those where cholera outbreaks had not been previously reported (e.g., Vubwi district).^5^^,^^6^ In Lusaka district, the first cholera case was reported in the Kanyama subdistrict, a high-density periurban area of the city, on October 15, 2023.^7^ The number of cases increased rapidly across other districts in Lusaka province, and the epidemic reached its peak on January 8, 2024. As of March 1, 2024, a total of 20,577 cases and 699 deaths were reported nationwide.^8^ During the outbreak, the Zambia National Public Health Reference Laboratory (ZNPHRL), operated by ZNPHI, conducted laboratory testing for suspected cholera cases as part of its mandate as a center of excellence in providing public health laboratory services. This study aimed to determine the antimicrobial susceptibility profile of *V. cholerae* isolated during the 2023–2024 outbreaks in Lusaka and other areas of Zambia by analyzing laboratory test results at ZNPHRL.

We tested a total of 2,384 stool samples that were collected from patients suspected for cholera at health care facilities in Eastern, Lusaka, and Luapula provinces in Zambia between January 1, 2023 and February 29, 2024 (Table 1). Although all provinces were eligible, samples were submitted only from Eastern, Lusaka, and Luapula provinces. Suspected cholera cases were defined as individuals of any age group who presented with three or more loose stools within 24 hours. Stool samples were submitted to ZNPHRL through the Provincial and District Health Offices for identification of* V. cholerae* using standard bacterial culture and serotyping. Culture-positive samples were subjected to antimicrobial susceptibility testing (AST) against panels of antibiotics, including ampicillin (ABPC), azithromycin (AZM), chloramphenicol (CAM), ciprofloxacin (CPFX), cotrimoxazole (SXT), and tetracycline (TC), through the Kirby–Bauer disc diffusion method using Muller Hinton agar. Selection of antibiotic discs and laboratory procedures in AST was based on the Clinical & Laboratory Standards Institute (CLSI) Guideline M45.^9^ Sensitivity to AZM was assessed only for samples collected from pediatric cases under 8 years old because the first-line antibiotic treatment option for this age group was AZM because of the contraindication of TC. The inhibition diameters were interpreted and categorized as being susceptible, intermediate, or resistant based on the CLSI Guideline M45.^9^

**Table 1:** Samples tested for cholera identification and antimicrobial susceptibility profile in Eastern, Luapula, and Lusaka provinces in Zambia between January 2023 and January 2024

Province	District	Year	Month	Number of Samples
Eastern	Chadiza	2023	January	1
	Chasefu	2023	January	1
	Chipangali	2023	January	6
			February	6
	Chipata	2023	January	1
			February	4
			July	4
			August	5
	Lundazi	2023	February	2
	Vubwi	2023	January	26
			February	5
			March	3
			April	2
			June	5
			July	1
			August	7
			September	3
			November	1
	Unknown	2023	January	1
Luapula	Chienge	2023	February	1
	Kawambwa	2023	February	1
	Mwansabombwe	2023	January	14
			February	41
	Nchelenge	2023	February	2
Lusaka	Chilanga	2024	February	1
	Chongwe	2023	November	3
			December	3
		2024	January	7
	Kafue	2024	January	2
	Luangwa	2023	November	7
		2024	January	3
	Lusaka	2023	October	420
			November	590
			December	569
		2024	January	449
			February	171
	Refunsa	2023	November	1
		2024	January	15

Total numbers of samples and their geographical origins and sample collection periods are indicated.

The secondary use of the laboratory test results, which were obtained as part of the public health services of ZNPHRL, for analysis and publication was approved by the National Health Research Authority (reference number NHRA-1453/09/08/2024).

A total of 549 samples (23.5%, *n* = 549 of 2,341) were identified to be culture positive for *V. cholerae* O1, including 117 (21.3%) of serotype Inaba and 401 (73.0%) of Ogawa. The median age (interquartile range) of the patients was 25 years old (15–36), and male gender was overrepresented (55.1%, *n* = 267 of 485). AST results were available for 431 (78.5%) of the 549 culture-positive samples. The proportion of *V. cholerae* being sensitive for TC, the first-line antibiotic treatment of adults, was 84.5% (*n* = 316 of 374) in Lusaka, whereas it was 100% among the tested samples in Eastern (*n* = 34 of 34) and Luapula (*n* = 20 of 20) provinces (Figure 1). The first TC-resistant *V. cholerae* was identified in epidemiological week 48 (EW48) of 2023, and it continued to be identified until EW8 of 2024 (Figure 2). One sample was resistant for AZM, the first-line antibiotic treatment of children, in Lusaka province (1.6%, *n* = 1 of 61), and no samples were shown to be resistant for AZM in other provinces (Figure 1). Sensitivity for CPFX, the second-line antibiotic treatment, was 81.8% (*n* = 260 of 318) in Lusaka province, whereas it was 100% among the tested samples in Eastern (*n* = 34 of 34) and Luapula (*n* = 20 of 20) provinces. CAM sensitivity was 100% in Luapula province (*n* = 20 of 20), 46.2% (*n* = 139 of 301) in Lusaka province, and 0 in Eastern province (*n* = 0 of 34). ABPC and SXT sensitivity was similar in all provinces, ranging from 0 to 2.8% (*n* = 9 of 318, SXT, Lusaka) (Figure 1).

**Figure 1.**
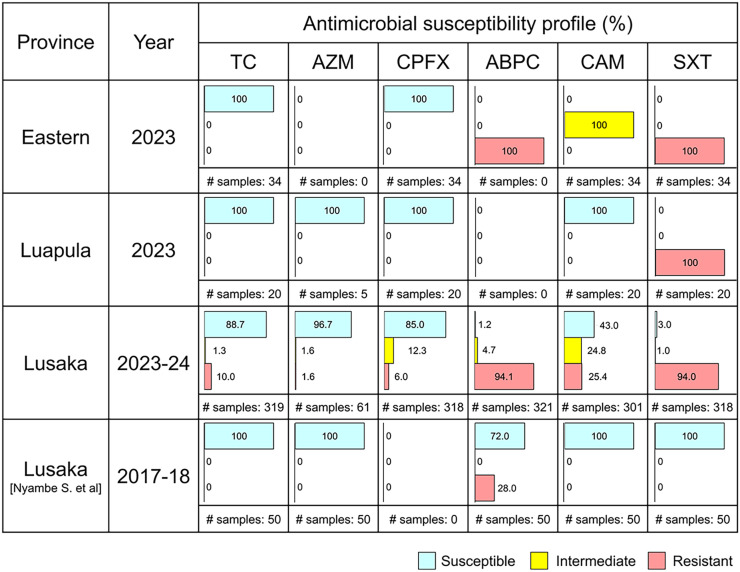
Antimicrobial susceptibility profile of *Vibrio cholerae* isolates identified in Eastern, Luapula, and Lusaka provinces from January 2023 to January 2024. Proportions (percentages) of isolates categorized as being sensitive, intermediate, and resistant (and not tested) to a panel of antibiotics, including tetracycline (TC), azithromycin (AZM), ciprofloxacin (CPFX), ampicillin (ABPC), chloramphenicol (CAM), and cotrimoxazole (SXT), are indicated with bar graphs. Data presented in a previous report from CDC^2^ are included as a reference.

**Figure 2.**
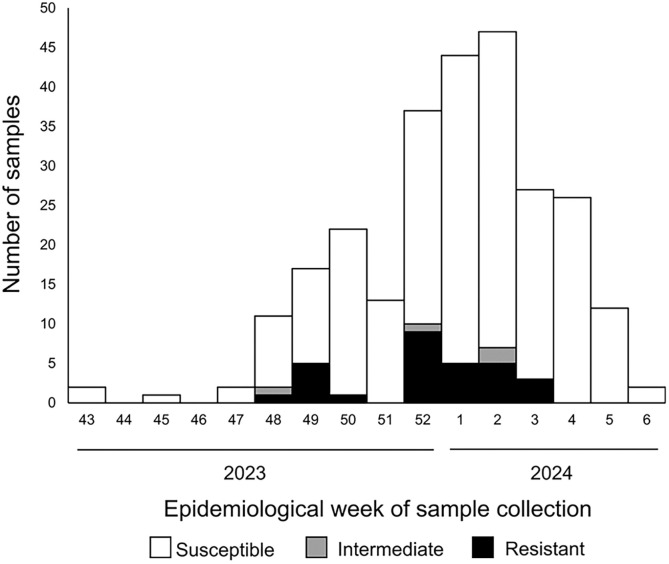
Temporal distribution of tetracycline-resistant isolates in Lusaka between October 2023 and February 2024. Numbers of samples identified with *Vibrio cholerae* categorized as being sensitive, intermediate, and resistant to tetracycline are indicated in the bar graph based on the epidemiological week of sample collection.

In this study, antimicrobial susceptibility profiles of* V. cholerae* for multiple antibiotics, including TC, AZM, CPFX, ABPC, CAM, and SXT, were determined for isolates identified in the outbreaks that occurred in Eastern, Luapula, and Lusaka provinces of Zambia between January 2023 and February 2024.

Notably, 10% of the isolates identified in Lusaka in 2023–2024 were found to be resistant to TC. Furthermore, the first AZM-resistant* V. cholerae* was identified in Lusaka in 2023–2024. During the previous major outbreak in Lusaka (2017–2018), it was reported that all isolates (*n* = 50) were 100% sensitive to TC, AZM, CAM, and SXT, and sensitivity to ABPC was 72%.^4^ These data suggest that resistant isolates to the first-line antibiotic treatment might have emerged or that the proportion of such resistant isolates increased significantly after the previous outbreak in Lusaka. Our findings were in line with the global trend of the increasing rate of TC-resistant strains.^10^ A previous report from the Democratic Republic of Congo (DRC) showed that loss of susceptibility to the antibiotics occurs over the years and that *V. cholerae* can acquire resistance to multiple antibiotics in those periods.^11^ In DRC, TC-resistant isolates were first identified in 2007, and it became one of the predominant types in the successive outbreaks after 2008 after acquiring additional resistance to CAM.^11^ This calls for the need to carefully monitor TC-resistant *V. cholerae* in future outbreaks in terms of its prevalence and acquiring additional resistance.

In addition, antimicrobial-resistant isolates for the first-line treatment options, TC and AZM, and the second-line treatment option, CPFX, were identified only in Lusaka province, whereas they were not identified in other provinces in 2023–2024. Multiple factors are assumed to be underlying in such geographical distribution patterns of resistant isolates, such as increased exposure to antibiotic treatment and transmission of resistant strains in Lusaka than in other provincial areas. In fact, previous studies documented high consumption of antibiotics, inappropriate use of antibiotics in health care facilities, and access to nonprescribed antibiotics in Lusaka district.^12^^,^^13^ A report from Bangladesh describes the emergence of TC- and erythromycin-resistant isolates in the capital Dhaka and its spread to provincial cities within a few years.^14^^,^^15^ Our results highlight the importance of improved prescribing practices in health care facilities and surveillance systems for such resistant strains in Lusaka and other parts of Zambia.

Limitations of this study include the potential effects of the larger number of samples during the outbreak in 2023–2024 compared with that in 2017–2018, which might have enhanced the surveillance capacity to capture a small proportion of resistant isolates. However, similar patterns of these isolates presenting reduced sensitivity to other antibiotics (e.g., ABPC, CAM, and SXT) support the possibility of increased resistance to TC and AZM. In addition, the study included samples collected from patients who visited health care facilities in 3 of 10 provinces in Zambia, which might have led to sampling bias.

Continuous efforts are required to prevent the emergence and spread of antimicrobial-resistant* V. cholerae* by careful and longitudinal monitoring of the regional antibiogram and reduction of antibiotic dependency (e.g., vaccine campaigns) in future cholera outbreaks in Zambia.

## References

[1] Finch MJ Morris JG Jr. Kaviti J Kagwanja W Levine MM, 1988. Epidemiology of antimicrobial resistant cholera in Kenya and East Africa. Am J Trop Med Hyg 39: 484–490. 3195696 10.4269/ajtmh.1988.39.484

[2] Centers for Disease Control and Prevention, 2024. Treating Cholera. Available at: https://www.cdc.gov/cholera/treatment/?CDC_AAref_Val=https://www.cdc.gov/cholera/treatment/antibiotic-treatment.html. Accessed October 14, 2024.

[3] Ministry of Health, Zambia, Zambia National Public Health Institute, World Health Organization, Global Task Force on Cholera Control, 2020. Risk Assessment and Mapping of Cholera Hotspots in Zambia: Review of Epidemiological Data from 2016 to 2019. Geneva, Switzerland: WHO.

[4] Sinyange N , 2018. Cholera epidemic—Lusaka, Zambia, October 2017–May 2018. MMWR Morb Mortal Wkly Rep 67: 556–559. 29771877 10.15585/mmwr.mm6719a5 PMC6048949

[5] Ministry of Health, Zambia, 2023. Press Release on Cholera Outbreak in the Country. Available at: https://www.moh.gov.zm/?p=3134. Accessed August 22, 2024.

[6] Ministry of Health, Zambia, 2024. Press Statement on the Cholera Situation in Zambia and Receipt of Oral Cholera Vaccines. Available at: https://www.moh.gov.zm/?p=3485. Accessed August 22, 2024.

[7] Lusaka Times, 2023. Cholera Outbreak in Lusaka: One Fatality and Swift Response. Available at: https://www.lusakatimes.com/2023/10/19/cholera-outbreak-in-lusaka-one-fatality-and-swift-response/. Accessed August 22, 2024.

[8] Ministry of Health, Zambia, 2024. Ministerial Statement on the Cholera Outbreak in Zambia. Available at: https://www.moh.gov.zm/?p=3549. Accessed August 22, 2024.

[9] Clinical & Laboratory Standards Institute, 2015. Methods for Antimicrobial Dilution and Disk Susceptibility Testing of Infrequently Isolated or Fastidious Bacteria, 3rd Edition. Available at: https://clsi.org/standards/products/microbiology/documents/m45/. Accessed August 22, 2024.

[10] Ahmadi MH, 2021. Global status of tetracycline resistance among clinical isolates of *Vibrio cholerae*: A systematic review and meta-analysis. Antimicrob Resist Infect Control 10: 115. 34362438 10.1186/s13756-021-00985-w PMC8343947

[11] Miwanda B , 2015. Antimicrobial drug resistance of *Vibrio cholerae*, Democratic Republic of the Congo. Emerg Infect Dis 21: 847–851. 25897570 10.3201/eid2105.141233 PMC4412219

[12] Ngoma MT Sitali D Mudenda S Mukuma M Bumbangi FN Bunuma E Skjerve E Muma JB, 2024. Community antibiotic consumption and associated factors in Lusaka district of Zambia: Findings and implications for antimicrobial resistance and stewardship. JAC Antimicrob Resist 6: dlae034. 38449513 10.1093/jacamr/dlae034 PMC10914457

[13] Masich AM , 2020. Antimicrobial usage at a large teaching hospital in Lusaka, Zambia. PLoS One 15: e0228555. 32040513 10.1371/journal.pone.0228555 PMC7010251

[14] Klontz EH Das SK Ahmed D Ahmed S Chisti MJ Malek MA Faruque AS Klontz KC, 2014. Long-term comparison of antibiotic resistance in *Vibrio cholerae* O1 and *Shigella* species between urban and rural Bangladesh. Clin Infect Dis 58: e133–e136. 24457344 10.1093/cid/ciu040 PMC3982837

[15] Islam MT , 2023. National hospital-based sentinel surveillance for cholera in Bangladesh: Epidemiological results from 2014 to 2021. Am J Trop Med Hyg 109: 575–583. 37580033 10.4269/ajtmh.23-0074 PMC10484282

